# Opioid and Cannabinoid Systems in Pain: Emerging Molecular Mechanisms and Use in Clinical Practice, Health, and Fitness

**DOI:** 10.3390/ijms25179407

**Published:** 2024-08-29

**Authors:** Carmine Secondulfo, Filomena Mazzeo, Grazia Maria Giovanna Pastorino, Antonella Vicidomini, Rosaria Meccariello, Francesca Felicia Operto

**Affiliations:** 1 Department of Medicine, Surgery and Dentistry “Scuola Medica Salernitana”, University of Salerno, 84081 Baronissi, Italy; csecondulfo@unisa.it (C.S.); graziapastorino@gmail.com (G.M.G.P.); anto.vicidomini96@gmail.com (A.V.); 2 Department of Economics, Law, Cybersecurity and Sports Sciences, University of Naples Parthenope, 80035 Nola, Italy; filomena.mazzeo@uniparthenope.it; 3 Child and Adolescent Neuropsychiatry Unit, “San Giovanni di Dio e Ruggi d’Aragona” Hospital, 84131 Salerno, Italy; 4 Department of Medical, Human Movement and Well-Being Sciences, University of Naples Parthenope, 80133 Naples, Italy; 5 Department of Science of Health, School of Medicine, University Magna Graecia of Catanzaro, 88100 Catanzaro, Italy

**Keywords:** pain, epigenetics, opioids, *Oprm1*, cannabinoids, CB1, CBD, clinical practice, health and fitness

## Abstract

Pain is an unpleasant sensory and emotional experience. Adequate pain control is often challenging, particularly in patients with chronic pain. Despite advances in pain management, drug addiction, overtreatment, or substance use disorders are not rare. Hence the need for further studies in the field. The substantial progress made over the last decade has revealed genes, signalling pathways, molecules, and neuronal networks in pain control thus opening new clinical perspectives in pain management. In this respect, data on the epigenetic modulation of opioid and cannabinoid receptors, key actors in the modulation of pain, offered new perspectives to preserve the activity of opioid and endocannabinoid systems to increase the analgesic efficacy of opioid- and cannabinoid-based drugs. Similarly, upcoming data on cannabidiol (CBD), a non-psychoactive cannabinoid in the marijuana plant *Cannabis sativa*, suggests analgesic, anti-inflammatory, antioxidant, anticonvulsivant and ansiolitic effects and supports its potential application in clinical contexts such as cancer, neurodegeneration, and autoimmune diseases but also in health and fitness with potential use in athletes. Hence, in this review article, we summarize the emerging epigenetic modifications of opioid and cannabinoid receptors and focus on CBD as an emerging non-psychoactive cannabinoid in pain management in clinical practice, health, and fitness.

## 1. Introduction

According to the International Association for the Study of Pain (IASP), pain is defined as an unpleasant sensory and emotional experience associated with actual or potential tissue damage or described in terms of such damage even in its absence [1]. This definition highlights that the concept of pain is not interchangeable with that of nociception, despite their close connection. Nociception refers to the process by which a harmful or potentially harmful stimuli are detected by specific receptors (which vary depending on the type of stimulus from the environment) and are transmitted to the central nervous system. Thus, nociception does not include the emotional and cognitive components that characterize pain [2]. Additionally, it is well known that the experience of pain varies depending on the individual, situation, mood, level of attention, and previous experiences [3]. Thus, the experience of pain cannot be simply reduced to a binary “all-or-nothing” mechanism but should be understood as a series of complex phenomena involving the integration and modulation of the basic signal. In this respect, opioid and cannabinoid receptors are widely co-distributed in the brain area involved in the processing of pain signalling but also in the spinal cord and peripheral tissues; as a consequence, they share key roles in nociception [4,5,6,7]. This has led to the clinical use of opioids and cannabinoids as analgesic drugs for the treatment of pain, but still controversies related to tolerance, addiction, or psychoactive effects occur [8,9,10,11]. Both systems are epigenetically modulated in health and disease [9,12] and the epigenetic modulation of gene expression of pro-nociceptive, anti-nociceptive, and inflammation-related genes has been recently reported [13,14].

Recently, attention has been focused on cannabidiol (CBD), a cannabinoid in the marijuana plant, that lacks psychoactive properties thus having promising results for the treatment of pain in several disease conditions [15,16], for physical health and fitness, and for possible use in athletes, due to a number of physiological, biochemical, and psychological effects potentially useful in improving performance, cell damage, and fatigue recovery, related to physical and cognitive exertion in sports, see [17,18,19] for recent review.

In this review article, we first provide a brief summary on the genesis and modulation of nociception in the nervous system; then, we summarize the emerging epigenetic modifications of opioid and cannabinoid receptor encoding genes in pain; lastly, we focus on CBD as an emerging non-psychoactive cannabinoid in pain management in clinical practice and discuss the use of CBD in health and fitness, and athletes.

## 2. Genesis and Modulation of Nociception in the Nervous System

Neurons responsible for nociception are pseudounipolar, with their cell bodies located in the dorsal root ganglia (DRG) of the spinal nerves (or in one of the sensory ganglia of the trigeminal nerve) and having two axonal projections that extend to the body’s periphery and the dorsal horn of the spinal cord (or the trigeminal nucleus; [20,21]).

Nociceptors transduce potentially harmful physical and chemical stimuli [22] and are divided into two types, based on their myelin sheath.

Aδ fibers: with a thin myelin sheath; Type I: respond to strong mechanical or chemical stimuli and, to a lesser extent, to high temperatures; Type II: respond primarily to thermal stimuli.

C fibers: completely unmyelinated C fibers are known to be polymodal, meaning they can respond to a wide variety of stimuli; some are even “silent”, typically becoming active only in the presence of concurrent inflammatory irritation [22,23]. This is an example of how the pain stimulus is modulated by various factors even at the peripheral level.

There are other differences among nociceptors: some C fibers release substance P (SP) and calcitonin gene-related peptide (CGRP) and respond to the nerve growth factor (NGF); other C fibers respond to the glial cell-derived neurotrophic factor (GDNF), neurturin, and artemin. Different nociceptors, moreover, respond to heat, cold, acidic milieu, or chemical irritants. Interestingly, while the peripheral terminal responds to ambient stimuli, both the peripheral and the central terminals respond to endogenous molecules such as pH, lipids, and neurotransmitters and can, therefore, be targets for analgesic treatments [23].

Various chemical mediators of inflammation, such as bradykinins, prostaglandins, histamine, interleukin (IL)-1β, tumor necrosis factor, and NGF significantly potentiate nociceptors by increasing neuronal excitability and decreasing nociceptor activation thresholds. These pathophysiological mechanisms underlie hyperalgesia (increased pain to a normally painful stimulus) and allodynia (pain in response to a normally non-painful stimulus) [24,25].

The NGF, primarily known for stimulating neuronal development, significantly modulates nociceptor signals at the peripheral level. It is produced by damaged and inflamed tissues [26,27], as well as by Schwann cells surrounding the nerves in case of direct damage [28]. NGF enhances nociception through various mechanisms, including signal transduction by multiple tyrosine kinases [29] and the facilitation of tetrodotoxin-resistant sodium currents while suppressing potassium outward currents, thereby increasing nociceptor activity [30]. Interestingly, genetic mutations for *NGF* or its receptor lead to pain insensitivity [31].

### 2.1. Modulation of the Signal in the Central Nervous System

Painful stimuli are transmitted to the central nervous system through the dorsal horn of the spinal cord, where nociceptors synapse with second-order neurons that then project via the spinothalamic and spinoreticulothalamic tracts. Specifically, Aδ and C fibers terminate in Rexed laminae I and II, establishing multisynaptic connections with non-painful somatosensory Aβ fibers. These ascending pathways primarily project to the thalamus, parabrachial nucleus, and amygdala [2,32].

Neurotransmitters used by nociceptors at spinal synapses include glutamate (acting on AMPA/kainate receptors), substance P (acting on Neurokinin 1 receptors, present in about 75% of nociceptors in lamina I), and CGRP (acting on a recently characterized heterodimeric Gs protein-coupled receptor). CGRP is involved in peripheral sensitization, being up regulated during inflammatory or neuropathic pain [33].

The peripheral and central nervous systems, though schematically divided, influence each other reciprocally. For instance, the facilitation of nerve signal genesis by inflammatory factors can elicit increased spontaneous activity centrally and reduce activation thresholds of wide dynamic range (WDR) neurons in the dorsal horn [24,34,35,36]. This can activate N-methyl-D-aspartate (NMDA) receptors, typically not involved in spinal nociceptive transmission, leading to neuronal hyperexcitability; thus, nociception can be modulated in long-term periods [37,38].

### 2.2. The Role of Brain Structures

The brain’s ability to modulate pain has long been suspected, as evidenced by observations of US soldiers in World War II [39], but experimental confirmations have come relatively recently. Pain is physiologically connected to peripheral nociceptive transmission, usually elicited by tissue damage, and includes a substantial emotional component, i.e., the unpleasantness associated with the pain experience. Ignoring either component overlooks an aspect of the entire issue [3].

These two aspects can be associated with two different components related to the brain’s processing of pain: a medial component (responsible for the emotional–motivational processing of pain) consisting of the medial thalamic nuclei, the anterior cingulate cortex, and the insular cortex, and a lateral component (related to the sensory–discriminative characteristics of pain), consisting of the primary and secondary somatosensory cortex, as well as the ventroposterolateral and medial thalamic nuclei [40]. These hypotheses have been demonstrated and supported by numerous studies, with some particularly emblematic cases reported: patients with lesions in the somatosensory cortex but with preserved medial component formations were unable to localize the sensation or describe its type (i.e., whether it was a warm/cold, burning/stinging sensation, etc.), but they felt a “clearly unpleasant” sensation vaguely coming from the limb where the stimulus was applied [41]. Conversely, patients suffering from intractable pain, after undergoing a cingulotomy (thus having a lesion in the medial component), reported immediate relief from the suffering associated with the pain, while still being able to distinguish the painful sensation, though it was devoid of its “unpleasant” characteristic [42].

### 2.3. Descending Pathways, Bidirectional Control, and DNIC

The discovery of the role of the periaqueductal gray (PAG) in nociception modulation has been crucial for understanding pain transmission modulation mechanisms. Tsou and Jang first demonstrated the profound antinociceptive effect of morphine microinjections in the rabbit PAG [43]. Subsequent studies have explored similar effects in animal models and humans, leading to the use of deep brain stimulation for treating intractable pain in selected patients [44].

The PAG receives opioidergic afferents from the anterior cingulate cortex, promoting its activity [45] and sending projections to pontine noradrenergic nuclei and serotonergic neurons in the rostroventromedial medulla (RVM) [46,47], exerting nociceptive inhibition via noradrenaline and serotonin [48].

The RVM communicates with the PAG, locus coeruleus, and thalamus, projects to the dorsal horn of the spinal cord and the dorsal trigeminal nucleus [49,50], and synapses with second-order interneurons that send ascending nociceptive projections, central to descending pain modulation [30].

The concept of Diffuse Noxious Inhibitory Controls (DNIC) emerged from observing that dorsal horn neuron activity (especially WDR neurons receiving Aβ and C fiber input) is inhibited by the concurrent application of a second nociceptive stimulus at an extra-segmental site [51,52]. Studies show that DNIC is integrated and coordinated at the dorsal reticular nucleus (DRt), connected to the PAG, RVM, thalamus, amygdala, and numerous cortical areas, sending fibers capable of modulating the pain stimulus to the spinal cord [53,54,55]. Each DRt neuron can send axons to multiple regions, functioning as a center capable of initiating multimodal pain modulation with continuous spinal–supraspinal–spinal feedback [56].

## 3. Opioid and Cannabinoid Receptors in Pain: Emerging Molecular Mechanisms

### 3.1. Opioid Receptors

Opioid receptors, which are G-protein-coupled receptors (GPCR), are expressed by neurons in both the central and peripheral nervous systems, as well as in neuroendocrine, ectodermal, and immune cells [57,58]. Three primary types of opioid receptors have been identified: mu (µ), delta (δ), and kappa (κ), also known as MOR, DOR, and KOR. These receptors are physiologically stimulated by endogenous opioid peptides, such as endorphins, dynorphins, and enkephalins. These molecules play a crucial role in modulating pain perception and influencing mood, behavior, and reward processing [59].

Current research, largely based on animal model studies, has elucidated distinct functions for each receptor type: µ receptors are primarily responsible for analgesia and the rewarding, dependence-inducing effects of exogenous opiates; δ receptors contribute to anxiolytic and antidepressant effects; and κ receptors, upon activation, induce psychotomimetic, hallucinogenic, and aversive responses, thus being associated with negative emotional experiences [60,61,62].

Opioid receptors are distributed throughout the nervous system, and exogenous opioid agonists can exert their effects at various levels, whether administered topically, epidurally, or systemically [63].

As previously described, DRG neurons activation is critical in the perception of both acute and chronic pain, and these cells express a great number of opioid receptors [64]. Peripheral activation of these receptors is responsible for a great part of the analgesic effect of exogenous opioids; lacking the notorious adverse effects of central opioid receptors stimulation, such as behavior alteration and potential addiction, there is a great interest both in clinical and research settings for a selective peripheral opioid receptor activation [65,66].

### 3.2. Cannabinoid Receptors

Classical cannabinoid receptors, namely CB1 and CB2, are GPCRs capable of mediating the effects of the over 100 phytocannabinoids found in the marijuana plant, *Cannabis sativa* L., [e.g., Δ^9^-tetrahydrocannabinol (Δ^9^-THC), Δ^8^-THC, cannabinol (CBN), CBD, cannabigerol (CBG), etc.)], endogenous cannabinoids [e.g., the main endocannabinoids anandamide (AEA) and 2-arachidonoyl glycerol (2-AG)], and synthetic cannabinoid agonists and antagonists [67]. While CB1 is largely distributed within the brain and at the periphery, the expression of CB2 is restricted to immune cells and in a few neurons within the brain but widespread distributed at the periphery [68,69]. In addition to endocannabinoids and classical cannabinoid receptors, the Na^+^ channel transient receptor potential vanilloid 1 (TRPV1), non-canonical receptors (e.g., GPR18, GPR55, and GPR119), and endocannabinoid-like compounds (e.g., N-acylethanolamines and 2-monoacylglycerols) hydrolyzing and biosynthetic enzymes, all together form the endocannabinoid system (ECS) [70,71]. This system has a recognized role in pain, mood, anxiety, depression, neurogenesis, neuroinflammation, synaptic plasticity, reward, cognition, learning, and memory. As a consequence, the ECS has received long lasting attention in pharmacology and clinical practice [71,72,73,74,75,76,77,78]. Nevertheless, cannabinoids, such as cannabis, hashish, and marijuana, that cause changes in mood and perception, euphoria, happiness, relaxation, deep sleep, and reducing anxiety, are considered drugs to use socially and recreationally.

As reviewed elsewhere, several studies indicate that the ECS regulates the nociceptive threshold, thus raising the possibility that the hypoactivity/inactivation of the ECS produces/prolongs chronic pain and hyperalgesia [6,7,79]. However, while studies in animal models provide substantial evidence that the exogenous modulation of the ECS hold considerable promise for the development of analgesic drugs, the challenge of translating this knowledge into clinically practice is quite controversial. Hence, the need for further studies in the field, to better understand molecular mechanisms, validate treatments, and improve clinical translation.

### 3.3. The Epigenetic Modulation of Genes Encoding for Opioid and Cannabinoid Receptors in Pain

**Figure 1:**
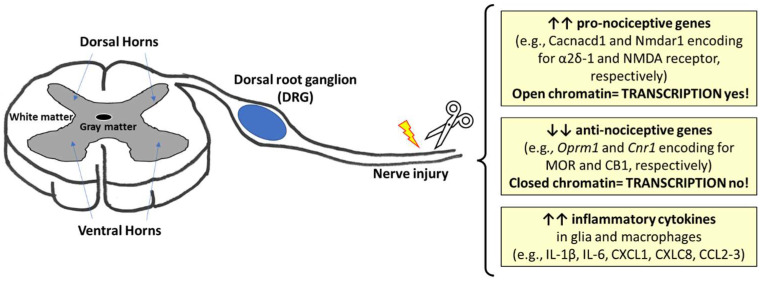
The main changes in gene expression reported in preclinical models of traumatic injury of peripheral nerves. ↑ increased expression; ↓ decreased expression.

Neuropathic pain is a clinical issue still difficult to treat. Changes in gene expression in peripheral sensory nerves and neurons as a consequence of injury have been reported (Figure 1) (for recent review [13,14]).

Nevertheless, few studies have investigated the epigenetic mechanisms at the basis of the increased expression of pro-nociceptive and the decreased expression of anti-nociceptive genes (e.g., genes encoding for α2δ-1, NMDA receptor, K^+^ channels, pannexin-1, opioid, and cannabinoid receptors) or those expressed in glia and macrophages encoding for inflammatory cytokines and chemokines in response to nerve injury [e.g., IL-1β, IL-6, and CXCL1, CXLC8, CCL2-3] [13,14]. Epigenetic changes affect gene expression without any effect on DNA nucleotide sequence and comprise DNA methylation at GpC sites; histone tail modifications—primarily acetylation (Hac) and methylation (Hmet) at specific lysine (K) residues; and the production of non-coding RNA (ncRNA). Epigenetic changes in DNA and chromatin architecture require the activity of epigenetic writers, erasers, and readers [80,81]. In preclinical studies, peripheral nerve injury induces differential and dynamic changes in DNA methylation status [82,83] and affects the expression rate of both non-coding RNA [9,14], and epigenetic machinery like the DNA methyltransferases, (DNMT), the epigenetic repressor methyl-CpG-binding domain protein 1 (MBD1) [84], 5-hydroxymethylcytosine converting enzyme (TET1) [85], arginine methylation enzymes (PRMT4, PRMT8, PRMT9), the methyltransferase EZH2, or the H3K9 methyltransferase G9a, the histone deacetylases (HDACs), and the histone acetylases (HATs) [14]; hence, the modulation of the epigenetic machinery by specific epigenetic activators or inhibitors represents a possible strategy in the modulation of injury-induced neuropathic pain. Focusing on opioid and cannabinoid receptors, in a rat model of chronic constriction injury (CCI) of the sciatic nerve, the trafficking of DOR in the brainstem nucleus raphe magnus (NRM) to pain-modulating neuronal synapses depends on the epigenetic upregulation of NGF by HDAC inhibitors [86]. DNMT inhibitors increased the expression of *Oprm1*, the gene encoding for MOR and MOR antagonism by naloxone exacerbated mechanical hypersensitivity induced by incision [87]. Consistently, the conditional knockout of *Ehmt2*, the gene that encodes for G9a, restores the physiological expression level of MOR and CB1 in DRG, thus restoring the analgesic effects of morphine [88], and potentiating the analgesic effects of CB1 agonists [89].

Furthermore, Viet et al. demonstrated [90] that in peripheral leukocytes the epigenetic regulation of *Oprm1* contributes to opioid tolerance in a cohort of cancer patients (n = 84) and demonstrated that in an animal model the reactivation of MOR expression in cancer cells inhibits mechanical and thermal hypersensitivity and prevents opioid tolerance [90]. 

Also, non-coding RNAs play a role in the epigenetic modulation of pain, drug addiction, and the healthy brain [9,91,92]. In particular, among the several microRNA capable of interacting with the 3′ untranslated region of *Opmr1* RNA, the *let-7* family of microRNA resulted as being critical when regulating MOR function in opioid tolerance [93]. Lastly, the METTL3-mediated m^6^A modification may occur in *Opmr1* RNA in a rat model of CCI [94].

Hence, epigenetic mechanisms should be useful in the treatment of pain to increase the analgesic efficacy of opioid- and cannabinoid-based drugs.

The main epigenetic changes in *Oprm1*, *Cnr1*, and *Cnr2* genes, respectively, encoding for MOR, CB1, and CB2, respectively, occurring in the DRG after traumatic nerve injury are summarized in Table 1.

## 4. Emerging Applications of *CBD* in Pain Management

### 4.1. Clinical Applications of CBD in Disease

In recent years, CBD, the second most prevalent active ingredient in cannabis, has been increasingly considered a safe alternative to other pharmacological therapies for various clinical conditions such as neurological condition, chronic pain, sleep disorders, and mood disorders [98].

CBD is present in both medicinal and fiber-type *C. sativa* plants (hepm), but, unlike Δ^9^-THC, it is completely non-psychoactive [99]. The safety of CBD has been further underlined by the World Health Organization (WHO) since pure CBD has been considered without abuse potential, even in high doses for children with drug-resistant epilepsy [100]. The most commonly reported side effects associated with CBD use are drowsiness and fatigue, dry mouth, nausea, reduced appetite, and diarrhea [101].

CBD can be found in different forms: pharmaceutical products, medical products, or wellness products/nutritional supplements. The only two pharmaceutical CBD-based products that have specific indications are expensive and unavailable for off-label use. Epidiolex is an oral solution containing 100 mg/mL of CBD, approved in the US and in 27 countries of the European Union for children and adults with Dravet and Lennox-Gastaut Syndrome. Nabiximols (Sativex^®^) is an oromucosal spray with 1:1 CBD:THC, approved in some jurisdictions for multiple sclerosis-associated spasticity and pain. Furthermore, in countries where medical cannabis is regulated, medical products with variable indications are available. These formulations may contain CBD with various quantities of THC [102]. Finally, many CBD products are marketed as dietary supplements/health products, raising more medical concerns as they are less regulated and potentially contaminated with chemicals, heavy metals, pesticides, and mycotoxins [102].

Preclinical evidence of CBD use suggests analgesic, anti-inflammatory, antioxidant, anticonvulsant, and anxiolytic effects. The principal molecular mechanisms responsible for these effects are the interactions with classical cannabinoid receptors (CB1, CB2); however, additional biological mechanisms are currently recognized. These mechanisms involve the interaction of CBD with other receptors and the start of intracellular signaling pathways, which further support its potential application in new clinical contexts such as cancer, neurodegeneration, and autoimmune diseases [15].

The clinical evidence is less strong, but several studies in recent years has focused on the use of CBD in the management of pain due to various clinical conditions. CBD can decrease nociception and reduce the frequency of painful symptoms by acting on the endocannabinoid system and involving neuro- and immuno-modulation at the central and peripheral levels [103].

CBD may be useful in treating chronic pain, a clinical condition defined as discomfort sensation that persists beyond 3–6 months or beyond the expected [104]. In light of the exponential increase in opioid use, there has been growing interest in alternative therapy for pain management, such as CBD, as potential treatment in order to decrease opioid prescribing. CBD-based therapy seems to be effective in reducing chronic pain in cancer and nonmalignant conditions (migraine, chronic pelvic pain, multiple sclerosis spasticity, neuropathic pain) and can decrease opioid prescriptions among patients with long-term conditions [105,106,107].

In particular, CBD has been proposed in several studies as a new treatment option in patients with chronic neuropathic pain; however, the authors have reached divergent conclusions on its efficacy [108,109].

In addition, CBD appears to alleviate symptoms of pain, sleep, and mood disorders in rheumatology patients, but sound clinical evidence is lacking [110]. Recent observational research has found an association between CBD use and improvements of pain symptoms in patients with arthritis and reductions in other medications; however, evidence from randomized controlled trials is lacking [111].

In very recent years CBD-based products have been suggested as a novel therapeutic option in patients with endometriosis, in which classic pharmacological therapies can often affect fertility; however, at the moment, there is a lack of conclusive evidence on the benefits [112].

Preliminary studies show that CBD-based therapy have demonstrated evidence for the management of migraines even though randomized, controlled trials are needed to support its clinical use. Currently, CBD is considered an integrative treatment added to traditional pharmacotherapy in patients with refractory migraines [113].

Finally, although CBD has only received FDA approval for Dravet Syndrome, Lennox Syndrome, and multiple sclerosis, CBD appears to show promise in many neurological conditions [114] such as trigeminal neuralgia, essential tremor, other forms of epilepsy, and neurodegenerative diseases such as Alzheimer’s [115] thanks to its neuroprotective and anti-inflammatory properties.

### 4.2. CBD in Health and Fitness, and Sport

The opioid and cannabinoid systems also influence other aspects of health and fitness with possible use in athletes due to physiological, biochemical, and psychological effects potentially capable of ameliorating several aspects related to physical and cognitive exertion in sports (see [17,18,19], for recent review). Table 2 summarizes the main features of opioids’ and cannabinoids’ effects, risks, and uses in sport.

By definition, *“health is a state of complete physical, mental and social well-being and not merely the absence of disease …. Fitness is an ability to execute daily functional activities with optimal performance, endurance, and strength to manage minimalist of disease, fatigue, stress and reduced sedentary behavior”* [116]. Understanding how exercise modulates these systems can inform strategies for pain management and improved well-being [117]. Exercise stimulates the release of endorphins and other endogenous opioids, which bind to opioid receptors, modulating pain signals [118]. Exercise can reduce inflammation by decreasing pro-inflammatory cytokines and increasing anti-inflammatory factors, thereby reducing pain [119]. However, studies suggest some evidence that women may be more susceptible to opioid addiction than men, although the reasons are not fully understood [120]. More research is needed to fully understand these differences and ensure optimal treatment for everyone [121]. Muscle contractions and adaptations can alter muscle and joint sensitivity, contributing to pain reduction [122]. Exercise activates descending pain inhibitory pathways in the brain, reducing pain perception and it can stimulate the release of endocannabinoids, which modulate pain perception. Moreover, exercise is increasingly recognized as a valuable adjunct to traditional pain management for conditions like lower back pain, fibromyalgia, and osteoarthritis [123].

Opioids are primarily used to relieve pain, but they can also produce other effects, such as drowsiness, constipation, and nausea. In sports, opioids are sometimes used to treat pain from injuries. However, they can also be addictive and have serious side effects, including overdose and death [124].

Cannabinoids act on cannabinoid receptors in the brain and body, and these receptors are involved in a variety of physiological processes, including pain perception, mood, appetite, and memory. In sports, some athletes use cannabinoids to treat pain, inflammation, and anxiety. However, cannabinoids can also impair coordination and reaction time, which can be a safety hazard in some sports [9].

Overall, the use of opioids and cannabinoids in sports is a complex issue and their use in sports is controversial. Some people believe that opioids are a necessary tool for pain management, while others believe that they are too risky. There are potential benefits and risks associated with both substances. Exercise offers a promising and often underutilized approach to pain management. By understanding the complex mechanisms underlying exercise-induced analgesia and tailoring exercise interventions to individual needs, healthcare providers can effectively incorporate physical activity into pain treatment plans [125]. More research is needed to determine the best way to manage pain and other conditions in athletes. Educating individuals about the risks of opioid and cannabinoid misuse is crucial for promoting healthy lifestyles.

Examining the potential benefits of combining exercise with other pain management interventions, such as anabolic steroids, physical therapy, and psychological therapies [126], there is some promise for CBD in sports and athletes are increasingly turning to CBD for its potential benefits on physical activity [19]. A recent randomized trial investigated the effects of 8 weeks of CBD and revealed a potential effect of CBD on power output but no changes in health-related fitness, physical activity, cognitive health, psychological wellbeing, and C-reactive protein [19]. In addition, consuming twice per day for 3.5 days a formulation containing CBD, CBG, beta caryophyllene, branched-chain amino acids (i.e., valine, leucine, and isoleucine), and magnesium citrate supports recovery from delayed onset muscle soreness on daily activities [127]. In another trial, CBD oil administered in capsules after a bout of exercise-induced muscle damage had no beneficial effects on perceived soreness and muscle function in untrained male subjects [128]. However, the limited number of trials in the field, involving non-athlete populations, highlights a gap between the marketing claims and the current scientific evidence for CBD and athletic performance [129].

Musculoskeletal pain is the most frequent in a traumatic context and pain management is a crucial issue for athletes who train and compete at the highest performance levels [9,130]. Studies have shown that CBD, via the modulation of the ECS, may be effective in reducing pain and inflammation caused by exercise [9]. Nevertheless, CBD goes beyond the anti-inflammatory, immunomodulatory, and antinociceptive properties, resulting as a promising modulator also for musculoskeletal regenerative medicine. In fact, as recently reviewed by Marquez Azzini [131], pre-clinical studies have revealed that CBD enhances cell proliferation and migration, especially in mesenchymal stem cells and has the ability to reverse or attenuate the hallmark of chronic musculoskeletal disorders. Interestingly, single CBD supplementation after intensive resistance training had small but significant effects on muscle damage biomarkers (i.e., blood serum concentrations of creatine kinase and myoglobin) and the recovery of squat performance after 72 h; this randomized controlled trial suggested the potential pro-regenerative effects of CBD supplementation after resistance training, but there is a need for further studies in the field [132].

Many athletes struggle with getting enough sleep, which is essential for recovery. CBD may help improve sleep quality by reducing anxiety and promoting relaxation. CBD may help reduce muscle soreness and inflammation after exercise, which can help athletes recover faster and get back to training sooner. In addition, CBD may help reduce anxiety and stress, which can improve athletic performance, reducing anxiety and stress [133]. Studies have suggested higher doses of CBD (around 10 mg/kg) might be needed to see an effect on exercise performance compared with the lower doses often marketed by some manufacturers. Moreover, long-term studies are needed to understand the full effects of daily CBD use on athletes and their performance. The evidence does not fully support daily use for everyone. 

The narcotics have been on the “prohibited list” from the World Anti-Doping Agency (WADA) since the list was created in 1967, despite the WADA removing CBD from the prohibited substance list in 2018. The WADA classifies cannabis, all phytocannabinoids, and synthetics as doping, except for CBD. For the WADA, in competition, all natural and synthetic cannabinoids are prohibited, e.g., in cannabis (hashish, marijuana) and cannabis products, synthetic cannabinoids that mimic the effects of THC and natural and synthetic THCs, with the exception of CBD [124,134]. Cannabis remains prohibited in competition by WADA and many other professionals and international organizations [135]. However, some sports organizations may still have their own rules regarding CBD use. Furthermore, the legality of CBD for athletes can vary depending on the sport and the organization governing the sport and athletes should always check with their governing body before using CBD products. While there is growing interest in CBD for fitness enthusiasts, the research on its direct impact on performance and addiction is inconclusive.

There is no evidence about any direct performance-enhancing effects of cannabis or CBD on athletes. Studies have not shown a clear benefit of CBD on enhancing physical performance or metrics like VO_2_ max (maximal oxygen consumption). While CBD is sometimes promoted for anxiety and stress relief, research on its impact on mental health in athletes specifically is inconclusive.

CBD is available in a variety of forms, including oils, tinctures, capsules, creams, and edibles. Athletes typically take CBD orally or apply it topically to sore muscles and joints. It is generally well-tolerated, but it can cause side effects such as diarrhoea, fatigue, and drowsiness [136]. It can also interact with certain medications. Athletes should be cautious and consult with a doctor before starting CBD to understand potential benefits and risks.

While CBD shows promise for athletes, the effects of THC on performance are generally negative. THC can negatively affect focus, reaction time, and decision-making, all of which are crucial for athletes. Also, increased heart rate and blood pressure can be detrimental during intense exercise. It is essential to approach cannabis use with caution and consider the potential risks and benefits carefully. THC can impair motor skills, affecting balance and agility, and impaired judgment and coordination can lead to accidents and injuries [137].

It is important to note that the research on CBD and sports is still in its early stages of confirming the potential benefits of CBD for athletes. Rigorous scientific research is essential to establish clear guidelines and recommendations. By investing in comprehensive research, we can develop evidence-based strategies for using these substances, safely and effectively, to enhance athletic performance and recovery.

## 5. Conclusions

The clinical use of opioids and cannabinoids as analgesic drugs for the treatment of pain raises the issue of drug addiction, overtreatment, or substance use disorders [8,9,10,11]. In the last years, pre-clinical studies on the “epigenetic modulation of pain” have provided insights into the molecular mechanisms of pain, revealing that the modulation of the epigenetic machinery by specific epigenetic activators or inhibitors may represent a possible strategy in the modulation of injury-induced neuropathic pain. In this respect, epigenetic mechanisms should be useful in the treatment of pain to increase the analgesic efficacy of opioid- and cannabinoid-based drugs.

In parallel, CBD appears to be a promising therapeutic agent to complement traditional medications for pain management. In light of its anti-inflammatory and neuroprotective potential, it also appears useful in the treatment of other neurological, psychiatric, and immunological conditions [138]. However, it must be considered that in clinical practice, it is rather difficult to evaluate the effect of CBD by separating it from the components contained in medical preparations, such as THC, flavonoids, and terpenes [139].

Similarly, the use of opioids and cannabinoids in health, fitness, and sports is a complex and controversial issue with potential benefits and risks associated with both substances. Nevertheless, there is some promise for the use of CBD in sports and athletes are consequently increasingly turning to CBD for its potential benefits on physical activity. More research is needed to determine the best way to manage pain and other conditions in athletes. Therefore, it would be necessary to conduct randomized and controlled studies that investigate the effect of pure CBD in order to better define its efficacy and safety to draw up guidelines that support the clinician in the management of the different conditions.

## Figures and Tables

**Table 1:** Epigenetic modulation of opioid and classical cannabinoid receptor genes in DRG following nerve injury.

Gene	Effects on Transcription	Epigenetic Modification	Reference
*Oprm1*	↓	↑ H3K9me2 ↑ H3K27me3 ↓ H3K4me3	[88]
*Oprm1*	↓	H3/H4 hypoacethylation	[95]
*Oprm1*	↓	↑ promoter DNA methylation via MBD1-dependent recruitment of DNMT3a	[84]
*Oprm1* and *Oprk1*	↓	↑DNA methylation via MBD1-dependent recruitment of DNMT3a for *Oprm1*	[96]
*Cnr1*	↓	↑ H3K9me2 at promoter region	[89]
*Cnr2*	↑	↑ H3K4me3 ↑ H3K9ac ↓ H3K9me2 ↓ H3K27me3 No effect on DNA methylation	[97]

↑ increase; ↓ decrease.

**Table 2:** The key differences between opioids and cannabinoids.

Feature	Opioids	Cannabinoids
Main effects	Pain relief	Varies depending on the specific cannabinoid (THC can be psychoactive, CBD is not)
Mechanism of action	Binds to opioid receptors in the central nervous system and body	Binds to cannabinoid receptors in the brain and body
Risks	Addiction, overdose, death	Impaired coordination and reaction time, potential for dependence
Use in sports	Pain management	Pain management, inflammation, anxiety (though prohibited by most anti-doping agencies)
